# Computationally-directed mechanical ventilation in a porcine model of ARDS

**DOI:** 10.3389/fphys.2025.1602578

**Published:** 2025-11-26

**Authors:** Michaela Kollisch-Singule, Andrea F. Cruz, Jacob Herrmann, Joshua Satalin, Sarah Satalin, Brian P. Harvey, Dorian LeCroy, George Beck, Mark Lutz, Jacob Charlamb, Joshua Kenna, Mark Baker, Gary F. Nieman, David W. Kaczka

**Affiliations:** 1 Department of Surgery, University of Arkansas Medical Sciences, Little Rock, AR, United States; 2 Division of Pediatric Surgery, Arkansas Children’s Hospital, Little Rock, AR, United States; 3 Department of Surgery, SUNY Upstate Medical University, Syracuse, NY, United States; 4 Department of Anesthesia, University of Iowa, Iowa City, IA, United States; 5 Roy J. Carver Department of Biomedical Engineering, University of Iowa, Iowa City, IA, United States; 6 Department of Clinical Sciences, Cornell University College of Veterinary Medicine, Ithaca, NY, United States; 7 ZOLL Medical Corporation, Chelmsford, MA, United States; 8 Department of Mathematics, SUNY Oswego, Oswego, NY, United States; 9 Department of Radiology, University of Iowa, Iowa City, IA, United States

**Keywords:** computational direction, mechanical ventilation, airway pressure release ventilation, acute respiratory distress syndrome, personalized

## Abstract

**Background:**

Despite the implementation of protective mechanical ventilation, ventilator-induced lung injury remains a significant driver of ARDS-associated morbidity and mortality. Mechanical ventilation must be personalized and adaptive for the patient and evolving disease course to achieve sustained improvements in patient outcomes. In this study, we modified a military-grade transport ventilator to deliver the airway pressure release ventilation (APRV) modality. We developed a computationally-directed (CD) method of adjusting the expiratory duration (T_Low_) during APRV using physiologic feedback to reduce alveolar derecruitment and tested this modality in a porcine model of moderate-to-severe ARDS.

**Methods:**

Female Yorkshire-cross pigs (n = 27) were ventilated using a ZOLL EMV+® 731 Series ventilator during general anesthesia and subjected to a heterogeneous Tween lung injury followed by injurious mechanical ventilation. Animals were subsequently ventilated for 6 hours under general anesthesia after randomization to one of three groups: V_T_6 (n = 9) with a tidal volume (V_T_) of 6 mL/kg and stepwise adjustments in PEEP and FiO_2_; V_T_10 (n = 9) with V_T_ of 10 mL/kg and PEEP of 5 cmH_2_O; CD-APRV group (n = 9) with computationally-directed adjustments in T_Low_ based on a nonlinear equation of motion to describe respiratory mechanics. Results are reported as median [interquartile range].

**Results:**

All groups developed moderate-to-severe ARDS and had similar recovery in lung injury, with all demonstrating final PaO_2_:FiO_2_ > 300 mmHg (V_T_6: 415.5 [383.0–443.4], V_T_10: 353.3 [297.3–397.7], CD-APRV: 316.6 [269.8–362.4]; p = 0.12). PaCO_2_ was significantly higher in the V_T_6 group compared with the CD-APRV group (59.3 [52.3–60.1] mmHg vs. 38.5 [32.7–52.2] mmHg, p = 0.04) but not significantly different from the V_T_10 group (47.5 [45.3–54.4] mmHg; p = 0.32 vs. V_T_6) despite having a significantly higher respiratory rate (30.0 [30.0–32.0] breaths/min) compared with V_T_10 (12.0 [12.0–15.0] breaths/min, p = 0.001) and CD-APRV (14.0 [14.0–14.0] breaths/min, p < 0.001) groups at the study end.

**Conclusion:**

We successfully implemented a computationally directed APRV modality on a transport ventilator, adjusting T_Low_ based on respiratory mechanics. This study demonstrated that CD-APRV can be safely used, with the advantage of guiding expiratory duration adjustments based on physiologic feedback from the lungs.

## Introduction

Acute respiratory distress syndrome (ARDS) is associated with high short- and long-term morbidity and mortality (Prescott et al., 2017). It is well-understood that mechanical ventilation, often required to support the severely injured lung, requires a careful and thoughtful approach to prevent a secondary ventilator-induced lung injury (VILI) (Slutsky and Ranieri, 2013). Although several lung-protective interventions such as low tidal volume ventilation and prone positioning have demonstrated initial successes in reducing mortality, further decreases in lung injury, morbidity, or mortality have not been observed (Bellani et al., 2016; Tonelli et al., 2014). To minimize VILI and achieve sustained reductions in morbidity and mortality, we hypothesize that mechanical ventilation needs to be both protective and personalized, adapting to changes in lung physiology over the course of the disease (Pelosi et al., 2021; Deans et al., 2005).

**FIGURE 1:**
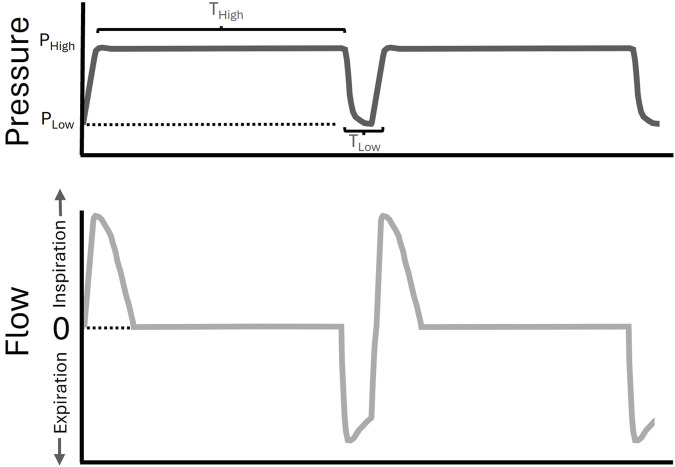
Airway Pressure Release Ventilation (APRV) waveform. A pictorial example of an APRV waveform (pressure versus time–upper panel in dark grey, flow versus time–lower panel in light grey). The inspiratory airway pressure (P_High_) is set for a duration (T_High_). The time (T_Low_) will determine the expiratory, or low, pressure (P_Low_) that is achieved, and was set by computational direction (CD) in the CD-APRV group.

Airway pressure release ventilation (APRV) is a mechanical ventilation modality that can be personalized and adaptive (Al-Khalisy et al., 2024), demonstrating efficacy in reducing the risk of ARDS in patients (Andrews et al., 2013) and in animal models (Kollisch-Singule et al., 2015; Roy et al., 2012; Roy et al., 2013). APRV uses prolonged inspirations to maximize alveolar recruitment over both short- and long-time scales (Kollisch-Singule et al., 2014; Nieman et al., 2023), with appropriate adjustments to expiratory duration (T_Low_) to prevent derecruitment (Figure 1). Ideally, T_Low_ should be long enough to maintain adequate ventilation (e.g., CO_2_ elimination) but sufficiently short to prevent derecruitment. The expiratory flow waveform must be precisely targeted to a temporal expiratory termination point in response to evolving lung pathology.

In this study, we developed a computationally-directed (CD) method for adjusting T_Low_ with APRV by considering physiologic feedback from the lung. We hypothesized that this CD strategy could be safely delivered, and we designed a lung injury model to test CD-APRV against both lower tidal volume (V_T_ 6 mL/kg) (Acute Respiratory Distress Syndrome et al., 2000) and larger tidal volume (V_T_ 10 mL/kg) ventilation.

## Materials and methods

All experiments were conducted with approval from the Institutional Animal Care and Use Committee (IACUC #332) and by the Animal Use and Care Office of the U.S. Department of Defense. The ARRIVE Guidelines 2.0 ensured proper reporting of methods, results, and discussion.

The commercially available ventilator (ZOLL EMV+ 731 Series transport ventilator) used in this study is not currently configured to deliver APRV. This study was, therefore, a joint industry and academic venture supported by a U.S. Department of Defense contract (W81XWH-20-1–0696), to integrate the APRV mode and test CD-APRV. However, the study is investigational, and authors from industry did not participate in any portions of the experimental design or data collection.

*Instrumentation:* Female Yorkshire-cross pigs (36.5 ± 5.3 kg) from a single farm (Keystone Mills, Romulus, NY) were acclimatized for 7 days prior to procedures. They were anesthetized with a continuous infusion of ketamine (9 mg/mL)/xylazine (0.009 mg/mL). Animals were continuously monitored for anesthesia adequacy ensuring a Stage III, Plane 2 level of anesthesia. A tracheostomy was performed with a 7.5-mm endotracheal tube (Harvard Apparatus) to establish a reliable airway and connected to the ZOLL EMV+ 731 Series transport ventilator, with baseline settings of: tidal volume (V_T_) 10 mL/kg, respiratory rate (RR) 12 breaths/min, positive end-expiratory pressure (PEEP) 5 cmH_2_O, and fraction of inspired oxygen (FiO_2_) 1.0. A central venous and Swan-Ganz catheter were placed in the bilateral external jugular veins for fluid and medication administration and cardiac monitoring. An ultrasound-guided femoral arterial line was placed for continuous monitoring of heart rate and blood pressure, as well as for arterial blood gas sampling.

*Heterogeneous lung injury model*: Pigs were transferred to a Dräger Evita v500 set to deliver continuous positive airway pressure (CPAP) of 18 cmH_2_O and FiO_2_ of 1.0. They were then subjected to a heterogeneous lung injury by instilling a 3% Tween-20 solution into the left and right diaphragmatic lobes (0.75 mL/kg per lobe) under bronchoscopic guidance, following a previously established protocol (Ramcharran et al., 1985). Tween deactivates pulmonary surfactant, which simulates this well-established component of ARDS pathophysiology (Slutsky and Ranieri, 2013). After withdrawal of the bronchoscope, animals received 10 min of injurious ventilation using APRV with P_High_ of 40 cmH_2_0, T_High_ of 2.5 s, P_Low_ of 0 cmH_2_O, and T_Low_ titrated to allow expiratory flow to reach 0 L/min.

*Mechanical ventilation protocol:* To standardize care and avoid any potential advantage associated with spontaneous breathing (Kollisch-Singule et al., 2019), animals in all groups were paralyzed with rocuronium (0.010–0.012 mg/kg/min) titrated to inhibit an inspiratory drive reflex. Following lung injury, an arterial blood gas was obtained, and the animal was transferred back to the transport ventilator (modified to deliver APRV) and ventilated according to one of three previously randomized groups. The V_T_6 group (n = 9) was ventilated with V_T_ of 6 mL/kg according to ARDS Network recommendations using the lower PEEP/higher FiO_2_ scale for stepwise adjustments (Acute Respiratory Distress Syndrome et al., 2000; Cannon et al., 2018). The V_T_10 group (n = 9) served as the Control and received V_T_ of 10 mL/kg, PEEP of 5 cmH_2_O, and RR of 12 breaths/min. The CD-APRV group (n = 9) had an inspiratory duration (T_High_) of 4.0 s, inspiratory pressure (P_High_) of 23 cmH_2_O, expiratory pressure (P_Low_) of 0 cmH_2_O, and CD adjustments in T_Low_, using a MATLAB program (MathWorks, Natick, MA) to determine expiratory duration. Pulmonary parameters were monitored continuously and recorded hourly in all groups. Pulmonary measurements were recorded from the Dräger Evita v500 for the V_T_6 and V_T_10 groups and from the ZOLL EMV+ 731 Series for the CD-APRV group. Inspiratory holds to establish a plateau pressure were avoided, since these would mimic recruitment maneuvers and potentially alter outcomes. The T_Low_ titration was also performed hourly and achieved by varying the value of the T_Low_ for three ventilatory cycles, from 0.2 s to 1.0 s, with increments of 0.05 s. Airway pressure (*P*) and flow ( $\dot{V}$ ) waveforms were measured with the monitor Florian (ACUTRONIC Medical System, Hirzel, Switzerland), sampled by an analog-to-digital converter (NI USB-6001, National Instruments, Austin, TX), low-pass filtered, and recorded in MATLAB. Respiratory system mechanics were described using the nonlinear equation of motion (Equation 1) (Kaczka et al., 2023; Bates, 2009; Bates et al., 2024):

$$P=R_{1}\dot{V}+R_{2}\dot{V}\left|\dot{V}\right|+E_{1}V+E_{2}V^{2}+P_{0}$$ (1)

where *V* denotes volume, *P*_0_ is the end-expiratory airway pressure, *R*_1_ and *R*_2_ are the resistance coefficients, and *E*_1_ and *E*_2_ are the elastance coefficients. The *R*_1_, *R*_2_, *E*_1_, *E*_2_, and *P*_0_ parameter estimates were obtained using multiple linear regression (Kaczka et al., 1995). The *E*_2_ vs. T_Low_ curve was fitted with a fourth-order spline, from which an “optimal” T_Low_ was obtained based on the maximal value of the spline second derivative (Supplementary Material S1). The rationale for the fourth order spline was to adequately characterize the point of maximum change in slope of the E2 vs. T_Low_ titration curve (Supplementary Figure S1), based on the curve’s second derivative. A fourth order spline allowed for a smooth second derivative of the curve, which allows T_Low_ to be maximized potentially at any value. With a third order spline, the second derivative would have been piecewise linear, with local maxima occurring only at the control points of the spline.

*Clinical monitoring and necropsy*: After 6 h of mechanical ventilation, the protocol was terminated, the animals were euthanized (pentobarbital 150 mg/kg), and necropsy was performed. The lungs were excised and inflated to 25 cmH_2_O, using stepwise increases in pressure for lung volume history standardization. Gross photos of the lungs were obtained at a 25 cmH_2_O inflation pressure. A tissue section from the apical (uninjured) and the right diaphragmatic (Tween-injured) lobes were excised. One segment from each tissue type was submerged in formalin for histopathologic analysis, and another was snap-frozen in liquid nitrogen for eventual mRNA analysis. Edema was assessed according to tissue wet-to-dry weight ratios.

*Histology*: The lung tissue was fixed in formalin and sent to HistoWiz Inc (Long Island City, NY) for standard hematoxylin and eosin staining. A quantitative histologic assessment was based on the image analysis of 540 photomicrographs (10 apical and 10 diaphragmatic for each animal), taken at high-dry magnification following a validated, unbiased, and systematic sampling protocol (Kubiak et al., 2010). Each photomicrograph was scored using a 4-point scale for each of five parameters: micro-atelectasis, white blood cells (WBCs) in the air space, red blood cells (RBCs) in the air space, edema, and capillary congestion (Supplementary Material S2). Though there is no distinct biomarker of derecruitment, histopathology combined with the clinical markers of compliance and oxygenation, and inflammatory cytokines were used as surrogate markers of atelectasis or derecruitment in this study.

*Inflammatory mediators:* Reverse transcription-quantitative polymerase chain reaction (RT-qPCR) was performed to investigate mRNA expression of key proinflammatory mediators: tumor necrosis factor-α (TNF-α), interleukin-6 (IL-6) and −8 (IL-8), and transforming growth factor-β (TGF-β) from the lung tissue. RNA extraction was performed using an RNeasy Mini kit (Qiagen, Germantown, MD) from RNA*protect* preserved lung tissue following the manufacturer’s instructions. The extracted RNA was resuspended in 40 μL of RNA-free water (Qiagen, Germantown, MD). Total RNA was quantified using a spectrophotometer at 260 nm, and purity was assessed by the optical density ratio at 260 nm/280 nm. Reverse transcription of RNA into cDNA was performed in a 20 μL total volume containing 1 μg of sample RNA using the High-Capacity cDNA Reverse Transcription kit (Thermo Fisher Scientific, Waltham, MA). Reverse transcription was performed for 10 min at 25 °C, followed by 120 min at 37 °C, and then 5 s at 85 °C. The solution was subsequently cooled to 4 °C. Quantitative RT-PCR was performed using the Step One Plus instrument (Thermo Fisher Scientific, Waltham, MA) and Fast Advanced Master Mix (Thermo Fisher Scientific, Waltham, MA) on all lung samples. Commercially available Taqman™ probes (Life Technologies, Carlsbad, CA) were used to amplify lung-related genes per the manufacturer’s instructions. Relative quantification of the target mRNA was performed using the comparative 2^−ΔΔct^ method with glyceraldehyde-3-phosphate dehydrogenase (*GAPDH*; Cat # Ss03374854_g1) as endogenous control.

### Statistics

A power analysis based on previously collected data using this injury model determined a sample size of 9 pigs per group to detect a difference in PaO_2_:FiO_2_ ratio with at least 90% power (Supplementary Material S2) (Ramcharran et al., 1985; Jain et al., 2017). Descriptive statistics are presented as median and interquartile range. For continuous variables, comparisons of treatment groups were conducted on post-treatment measurements collected at the final time point of the study, using either analysis of variance (ANOVA) with *post hoc* analysis performed using the Tukey-Kramer method to adjust for multiple comparisons, or the Kruskal–Wallis test with *post hoc* analysis conducted using the Dwass, Steel, Critchlow-Fligner method to adjust for multiple comparisons. ANOVA was implemented when the normality assumption was met (as evaluated using the Shapiro-Wilk test of the residuals and Q-Q plots). For significant results, simultaneous 95% confidence intervals were produced using the Tukey-Kramer adjustment for the parametric case, and Bonferroni corrected simultaneous 95% confidence limits for the location shift were reported for the nonparametric case. Histologic injury scores were modeled as the dependent variable and treatment type as the independent variable while controlling for the factor of the different scorers and the dependency due to multiple samples taken on the same subjects post-treatment. Histologic injury scores are reported as mean and standard deviation.

Due to the exploratory nature of this pilot study, adjustments for multiple comparisons were only made during *post hoc* analyses. Since the mild acute lung injury and treatment types delivered to subjects were not expected to be a potential cause of death in subjects, observations of those subjects that did not survive to the end of the study and did not have a final time point were treated as missing at random for the relevant analysis conducted on the final time point. This study compared a pressure-regulated (CD-APRV) against two volume-regulated (V_T_6 and V_T_10) modes, making a statistical comparison of the differing dependent variables (tidal volume and PEEP in CD-APRV and plateau pressure in V_T_6 and V_T_10) less relevant and was therefore selectively excluded. A nominal significance level of 0.05 was used in all testing, and statistical analyses were performed using SAS (V9.4). Descriptive statistics were produced using Excel (2016, Microsoft).

Animals were randomized to ventilation groups (Supplementary Material S1). The investigators were not blinded to the allocated groups during the experiments because ventilation adjustments were necessary during the study. Two investigators (JS, GFN) performed the histologic analysis and were blinded to the treatment groups and samples. An independent statistician performed the data analysis to mitigate potential bias.

## Results

### Pulmonary parameters

**FIGURE 2:**
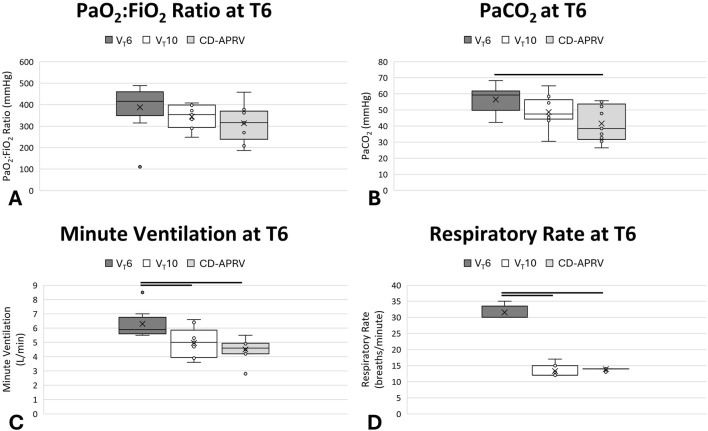
Pulmonary parameters. Oxygenation, represented by PaO_2_:FiO_2_ ratio **(A)**, PaCO_2_ **(B)**, minute ventilation **(C)** and respiratory rate **(D)** in animals ventilated with 6 mL/kg tidal volumes (V_T_6; medium grey), 10 mL/kg tidal volumes (V_T_10; white) and computationally directed APRV (CD-APRV; light grey) at the final timepoint (T6). Median represented by black line within each box. The bottom of the box marks the first quartile (25th percentile) and the top marks the third quartile (75th percentile). X denotes the mean and individual data points are represented by small circles. Statistical comparisons were made at the study endpoint (T6) with black lines representing p < 0.05 versus V_T_6.

Tween injury established a moderate-to-severe ARDS with a decrease in the PaO_2_:FiO_2_ ratio in the three groups directly after injury (V_T_6: 415.5 [383.0–443.4] mmHg; V_T_10: 353.3 [297.3–397.7] mmHg; CD-APRV: 316.6 [269.8–362.4] mmHg; Supplementary Table S2). Oxygenation recovered over the ensuing 6 h, such that all groups had a final average PaO_2_:FiO_2_ ratio above 300 mmHg (p = 0.12) with varying FiO_2_ by the study end (p = 0.03), but that was not significant between groups in *post hoc* analysis (p > 0.05 for all comparisons; Figure 2A). Ventilation was initially impaired in the V_T_6 group and terminated with a non-significant increase in PaCO_2_ (59.3 [52.3–60.1] mmHg) as compared with V_T_10 (47.5 [45.3–54.4] mmHg; p = 0.32 vs. V_T_6) and a significant increase relative to CD-APRV (38.5 [32.7–52.2] mmHg; p = 0.04 vs. V_T_6; 95% CL for V_T_6 vs. CD-APRV [0.1,28.8]; Figure 2B): by the study end. While tidal volumes for the V_T_6 (6.0 [5.8–6.0] mL/kg) and V_T_10 (10.0 [9.9–10.1] mL/kg) groups were set, tidal volumes in CD-APRV (10.4 [9.9–11.0] mL/kg) are dependent on underlying lung compliance such that there was a relative difference in tidal volumes among the three groups. To increase minute ventilation in the V_T_6 group to compensate for rising PaCO_2_ (Figures 2B,C), the respiratory rate required adjustment such that it was significantly higher (30.0 [30.0–32.0] breaths/min) than both the V_T_10 (12.0 [12.0–15.0] breaths/min; p = 0.001 vs. V_T_6; 95% CL for V_T_6 vs. V_T_10: [15.0,20.0]) and CD-APRV (14.0 [14.0–14.0] breaths/min; p < 0.001 vs. V_T_6; 95% CL for V_T_6 vs. CD-APRV [16.0,19.0]; Figure 2D; Supplementary Table S2): groups by the study end.

Peak pressure was significantly higher in CD-APRV (29.2 [28.9–29.4] cmH_2_O) as compared with V_T_6 (23.4 [19.0–26.9] cmH_2_O; p = 0.003 vs. CD-APRV; 95% CL for V_T_6 vs. CD-APRV: [-11.7,-1.4]) and V_T_10 (23.5 [22.5–25.3] cmH_2_O; p = 0.001 vs. CD-APRV; 95% CL for CD-APRV vs. V_T_10: [3.2,7.3]) by the final time point (Supplementary Table S2). The plateau pressure in the CD-APRV group (22.0 [21.8–22.7]) was comparable to the peak pressures in the V_T_6 and V_T_10 groups. PEEP was adjusted according to the ARDSnet protocol in the V_T_6 group and was therefore higher (10.0 [8.0–10.0] cmH_2_O) than the V_T_10 group (5.0 [5.0–5.0] cmH_2_O) in response to the low PaO_2_:FiO_2_ ratio. While the P_Low_ was set to 0 cmH_2_O in CD-APRV, the computational direction set the T_Low_ short enough that this pressure was never achieved. The CD T_Low_ was 0.49 [0.49–0.49] s following injury and 0.51 [0.50–0.51] s by the experiment end. The transport ventilator was not configured to measure this T_Low_-controlled PEEP and thus is not reported. The V_T_6 group had a significantly higher mean airway pressure (11.3 [8.5–12.7] cmH_2_O) as compared with the V_T_10 group (7.8 [7.6–8.4] cmH_2_O; p = 0.03 vs. V_T_6; 95% CL for V_T_6 vs. V_T_10: [0.1,6.3]), which was likely secondary to a combination of the increased PEEP and respiratory rate in the V_T_6 group. The CD-APRV group had the highest mean airway pressure (20.4 [20.1–20.6] cmH_2_O; p = 0.002 vs. V_T_6; 95% CL for V_T_6 vs. CD-APRV: [-12.4,-6.2] and p = 0.001 vs. V_T_10; 95% CL for CD-APRV vs. V_T_10: [11.6,13.0]), as anticipated given the increased duration at the inspiratory pressure.

### Hemodynamics and mortality

Heart rate was similar across groups (V_T_6: 90 [78–99] beats/min; V_T_10: 90 [78–96] beats/min; CD-APRV: 105 [78–118] beats/min; p = 0.74), however the mean arterial pressure was lower in the CD-APRV group (77 [67–87] mmHg) as compared with the V_T_6 (99 [93–109] mmHg; p = 0.005 vs. CD-APRV; 95% CI for CD-APRV vs. V_T_6: [-35.2,-6.1]) and V_T_10 groups (86 [81–106] mmHg; p = 0.07 vs. CD-APRV; Supplementary Table S3). Pulmonary artery pressure was similar among groups (V_T_6: 30.5 [24.8–36.3] mmHg; V_T_10: 25.5 [24.0–26.3] mmHg; CD-APRV: 29.0 [26.5–33.5] mmHg; p = 0.41). V_T_10 was associated with a lower pulmonary capillary wedge pressure (12.0 [11.0–12.0] cmH_2_O) as compared with the V_T_6 (14.0 [12.5–16.5] cmH_2_O; p = 0.39 vs. V_T_10) and CD-APRV groups (15.0 [15.0–15.8] cmH_2_O; p = 0.009 vs. V_T_10; 95% CL for CD-APRV vs. V_T_10: [1.0,7.0]). Animals had similar total fluid requirements among groups (p = 0.43), but the CD-APRV group had a lower cumulative urine output compared with the V_T_6 and V_T_10 groups (p < 0.05 vs. V_T_6 and V_T_10 at T6; 95% CI for CD-APRV vs. V_T_6 [-818.8,-44.7] and 95% CI for CD-APRV vs. V_T_10 [-812.1,-180.2]) such that the CD-APRV group had a higher fluid balance (p = 0.03 among groups with p < 0.05 vs. V_T_10 at T6; 95% CL for CD-APRV vs. V_T_10 [89.0,1187.0]; Supplementary Table S3). A total of 27 pigs were included. Only one animal in the V_T_6 group had an early death 4 hours after injury due to a sudden cardiovascular collapse that was not responsive to epinephrine injection. There were no other adverse events, and no animals were excluded from the study or analysis. No other animals in the groups had a hypotensive episode or required vasopressors for support.

### Inflammatory mediators

**FIGURE 3:**
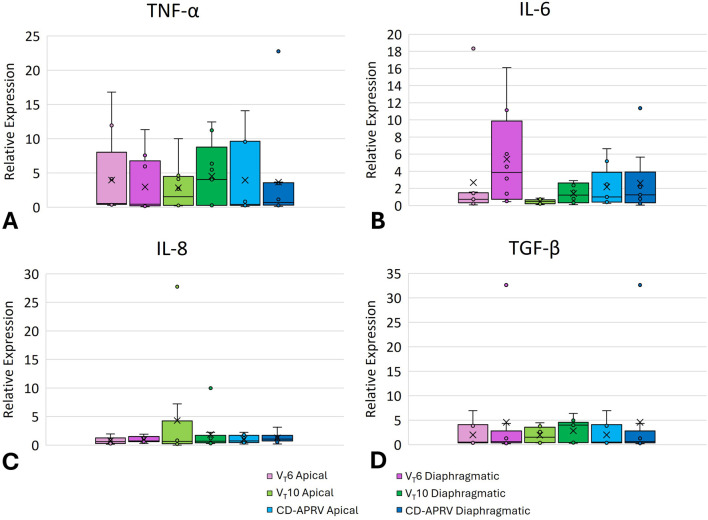
Pulmonary inflammatory mediators. Box plot of lung mRNA expression of TNF-α **(A)**, IL-6 **(B)**, IL-8 **(C)**, and TGF-β **(D)** from 6 mL/kg tidal volume (V_T_6 - purple), 10 mL/kg tidal volume (V_T_10 - green) and computationally directed APRV (CD-APRV - blue) ventilation strategies. Median represented by black line within each box. The bottom of the box marks the first quartile (25th percentile) and the top marks the third quartile (75th percentile). X denotes the mean and individual data points are represented by small circles. Apical is represented by lighter colors and diaphragmatic with darker colors.

All three groups demonstrated increases in lung inflammatory mediators TNF-α, IL-6, IL-8 and TGF-β (Figure 3). All groups showed an increase in TNF-α in both the apical and diaphragmatic lobes (Figure 3A; p = 0.72 for apical and p = 0.68 for diaphragmatic). The V_T_10 group demonstrated relatively low IL-6 expression as compared with V_T_6 and CD-APRV groups (Figure 3B; p = 0.22 for apical and p = 0.17 for diaphragmatic) but relatively higher expression of IL-8 (Figure 3C; p = 0.86 for apical and p = 0.63 for diaphragmatic). IL-6 expression was relatively higher in the diaphragmatic lung tissue in all three groups. TGF-β demonstrated similar expression among groups (Figure 3D; p = 0.70 for apical and p = 0.50 for diaphragmatic).

### Histopathology

**FIGURE 4:**
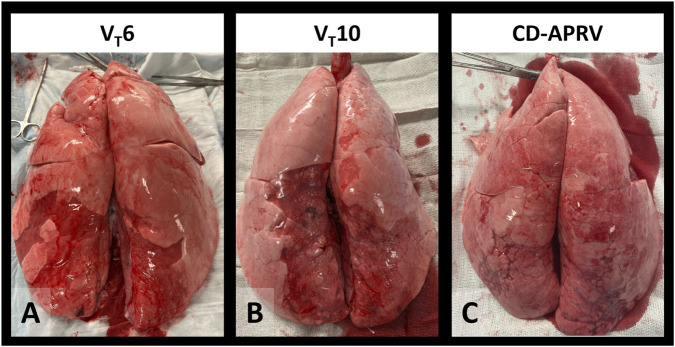
Gross lung pathology. Excised lungs of animals ventilated with 6 mL/kg tidal volumes (V_T_6 – **(A)**, 10 mL/kg tidal volumes (V_T_10 - **(B)** and computationally directed APRV (CD-APRV - **(C)**.

Wet-to-dry weights were higher in the diaphragmatic lobes (V_T_6: 7.2 ± 1.3; V_T_10: 7.9 ± 1.5; CD-APRV: 7.3 ± 1.0) than the apical lobes (V_T_6: 6.8 ± 2.8; V_T_10: 5.7 ± 0.5; CD-APRV: 5.9 ± 0.7) but not significant among groups (p = 0.16 in apical and p = 0.47 in diaphragmatic regions). Gross lung pathology revealed inflammation in all three groups, consistent with Tween injury. The lung apices were well-recruited and uninjured in all groups, whereas the dependent, diaphragmatic lobes demonstrated atelectasis, though this was less pronounced in the CD-APRV group (Figure 4).

**FIGURE 5:**
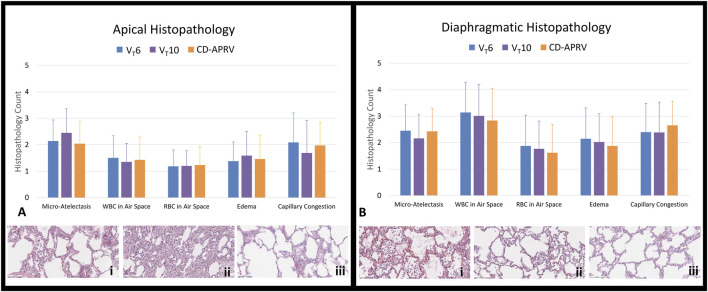
Lung histopathology. Histopathology (20x) of excised lungs of animals ventilated with 6 mL/kg tidal volumes (V_T_6; blue), 10 mL/kg tidal volumes (V_T_10; purple) and computationally directed APRV (CD-APRV; orange). Injury scoring was performed for micro-atelectasis, white blood cell (WBC) in the air space, red blood cell (RBC) in the air space, edema, and capillary congestion. **(A)** Histopathology scoring and representation of the apical lobes with V_T_6 revealing WBCs in the air space with capillary congestion (i), prominent micro-atelectasis in V_T_10 (ii), and WBCs in the air space with capillary congestion in CD-APRV (iii). **(B)** Histopathology scoring and representation of the diaphragmatic lobes with V_T_6 demonstrating edema and prominent WBCs in the air space (i), V_T_10 with edema and scattered WBCs in the air space (ii), and CD-APRV with capillary congestion (iii). n = 9/group. Error bars represent SD. There was no significant difference among groups.

The diaphragmatic lobes had relatively higher histologic injury scores compared to the apical lobes, consistent with the targeted injury (Figure 5). Histopathology of the apical lobes revealed increased capillary congestion and WBCs in the air space in the V_T_6 (Figure 5Ai) and CD-APRV groups to a lesser extent (Figure 5Aiii). Increased micro-atelectasis and edema were present in the V_T_10 group but with decreased capillary congestion (Figure 5Aii). Histopathology of the diaphragmatic lung lobes demonstrated increased WBC and RBC in the air space and edema in the V_T_6 group compared to V_T_10 and CD-APRV (Figure 5Bi). There were no significant differences in individual histopathologic parameters among groups (p > 0.05 for all comparisons across treatments). The V_T_10 group demonstrated modest edema and alveolar WBCs (Figure 5Bii), with more prominent capillary congestion observed in CD-APRV (Figure 5Biii). Inter-rater reliability between the two histologic scores was modest, with an average difference of 0.39 ± 0.35 among all parameters and lung regions (Supplementary Table S1).

## Discussion

This study found that computational direction can be used to adjust T_Low_ in APRV using physiologic feedback from the lung. While not specifically studied in this project, such a parameter has the potential to be automated into any ventilator that is equipped to deliver the APRV mode. A higher tidal volume (V_T_ 10 mL/kg) group was incorporated in this study to serve as a comparator group with tidal volumes matching those expected in CD-APRV, with both groups demonstrating improved ventilation compared with the V_T_6 group. Computational direction has the potential to provide a personalized approach to mechanical ventilation that can recommend T_Low_ adaptations informed by lung physiology throughout an evolving disease course. There is potential for relevant clinical translation of our technique, both for military-grade transport ventilators used in pre-hospital settings and austere environments, as well as for bedside mechanical ventilators. This computational direction of APRV highlights a strength of this study, which is the use of an innovative and physiologically grounded approach using nonlinear modeling. The experiment was designed using a previously validated lung injury model (Jain et al., 2017) that recreates the clinical features and physiological, biological, and pathological changes that are recommended for a translational model (Matute-Bello et al., 2008). There are several limitations to this study that deserve attention, including the protocol duration, which may not have been sufficient to detect pathophysiologic and inflammatory differences among groups. Though the study was designed with appropriate comparator groups, the use of two volume-targeted modes against a pressure-regulated mode created a challenge in precisely comparing pressure measurements, such as PEEP and plateau pressure across modes. This is a notable limitation that warrants further study evaluating CD-APRV against other pressure-regulated modes. The study was not blinded given the nature of the mechanical ventilator adjustments, though this was mitigated to the greatest extent with pig randomization, blinded histopathology scoring and the use of an independent statistician.

Ventilation-induced lung damage can occur even in lungs without a predisposed injury (Gajic et al., 2004; Emr et al., 2013) but can have even more severe consequences in patients with regionally higher micro-stresses and -strains in the setting of already damaged lung (Bates and Smith, 2018). Furthermore, VILI can be established within just a few hours (Dreyfuss et al., 1988; Webb and Tierney, 1974), and it is well-accepted that early protective mechanical ventilation can modify outcomes and survival in ARDS (Needham et al., 2015; Fuller et al., 2017). Determining methods of setting and dynamically adjusting ventilation parameters to minimize injury is therefore critical to protecting patients and improving outcomes. Airway pressure release ventilation is a mode of ventilation that has been proposed to help mediate VILI with pressure and uncoupled time settings, allowing for titration according to patient characteristics, disease pathophysiology, and evolution over time (Al-Khalisy et al., 2024). With essentially infinite ways of setting APRV, the very aspect that makes APRV attractive also offers the potential for harm if not set purposefully. The frequently published method of setting APRV, time-controlled adaptive ventilation (TCAV), advocates for setting T_Low_ according to the expiratory flow (Habashi, 2005; Nieman et al., 2020). The expiratory flow angle changes are congruent with lung compliance. As an example, the expiratory flow angle becomes more obtuse with improving compliance and more acute with decreasing compliance (Ramcharran et al., 2024). Shortening the T_Low_ to accommodate decreasing lung compliance and prevent recruitment/derecruitment at expiration could be considered analogous to increasing PEEP in a patient with increasing driving pressures and decreased lung compliance (Amato et al., 2015).

Despite the publicized TCAV methodology, alternate methods of setting APRV have been selected (Lutz et al., 2024), with variations including targeting tidal volumes (Zhou et al., 2017), setting P_Low_ > 0cmH_2_O (Zhou et al., 2017; Wrigge et al., 2001; Varpula et al., 2004), and lengthening T_Low_ (Wrigge et al., 2001; Varpula et al., 2004; Manjunath et al., 2021; Kucuk et al., 2022), some with poor outcomes. This variation highlights the imminent need for an informed but, ideally, personalized strategy. Each of the individual settings (P_High_, T_High_, P_Low_, T_Low_, FiO_2_) requires careful attention and has the potential to benefit from computational direction. T_Low_ represents a particularly important ventilation parameter for maintaining alveolar stability at expiration and was specifically selected for study. Setting the T_Low_ too long allows for alveolar derecruitment, where recruitment/derecruitment can determine physiologic recovery but must be balanced against hypercapnia that might result if set too short (Gaver et al., 2025). Thus, minimizing recruitment/derecruitment by optimizing T_Low_ represents an important target for informed mechanical ventilation.

CD-APRV was studied using a transport ventilator, assuming that informed device settings based on physiologic feedback would not only be beneficial but may also allow caregivers saturated with other tasks to be attentive to other medical needs. However, our study was not conducted in a transport or pre-hospital setting. Rather, this proposed computational direction could be applied to any ventilator that is capable of delivering APRV. Furthermore, the CD established in this study was not applied autonomously, though this represents a future direction for this project. Autonomous control of mechanical ventilation has precedent and is feasible and effective with FiO_2_ adjustments (Johannigman et al., 2009). Eventual integration of autonomous mechanical ventilation is attractive, as it may result in better adherence to lung-protective protocols. Even low tidal volume ventilation, which has gained widespread acceptance (Acute Respiratory Distress Syndrome et al., 2000) is inconsistently applied to patients (Weiss et al., 2016; Poole et al., 2017). One study evaluated three trials and found that compliance with low tidal volume ventilation ranged from 20%–39% (Gaver et al., 2025) while another demonstrated that less than 20% of the studied patients with ARDS received low tidal volume ventilation (Weiss et al., 2016). The future direction of this project will be to proceed with further preclinical testing in a longer duration model with more severe lung injury, and to improve the software to allow for more frequent autonomous adjustments that could then be integrated into mechanical ventilators supporting patients. T_Low_ adjustments may be required often, particularly in the acute stages of resuscitation and inflammation, as lung injury is evolving. We anticipate that CD-T_Low_ assessments and adjustments could be made as often as hourly, but also potentially triggered by noted changes in compliance, to prevent lung derecruitment.

The phenotype of ARDS may also influence the physiologic response to changes in ventilation. For example, patients with extrapulmonary ARDS tend to be more PEEP-responsive, compared with patients with pulmonary ARDS (Gattinoni et al., 1998). Additionally, patients with underlying obstructive lung pathology may not benefit from a shorter T_Low_ due to the concern for air-trapping. Thus, the ability to use feedback acquired directly from the lung to inform ventilator adjustments represents a forward view for mechanical ventilation. Patients with extrapulmonary ARDS often benefit from ventilation with a higher mean airway pressure, which is one advantage of APRV given the extended inspiratory duration (T_High_) at the higher airway pressure (P_High_) (Kollisch-Singule et al., 2015). One of the disadvantages of a higher mean airway pressure and increased positive intrathoracic pressure, however, is the altered vascular driving pressure from increased right ventricular afterload and decreased preload (Corp et al., 2021). Despite similar fluid resuscitation, our CD-APRV group had a significantly higher mean airway pressure, with expected decreases in mean arterial pressure and urine output, compared with the V_T_10 and V_T_6 groups. This should be an important consideration when using APRV in critically ill patients, who may be intravascularly volume depleted, and require fluid resuscitation before initiating ventilation with higher mean airway pressures.

In this acute (6 h) model of moderate-to-severe ARDS, the application of CD-APRV was found to be non-inferior to V_T_10 and V_T_6, with similar oxygenation and FiO_2_ requirements. A higher tidal volume (V_T_10) was selected for comparison but did not demonstrate a significant decrease in oxygenation. Instead, the V_T_10 and CD-APRV groups demonstrated improved CO_2_ elimination compared with the V_T_6 group based on arterial blood gases. Hypercapnia in low tidal volume ventilation is well-described due to the concomitant decrease in minute ventilation (Fuchs et al., 2011), and so the respiratory rate was increased following the ARDS Network protocol, but did not influence oxygenation (Acute Respiratory Distress Syndrome et al., 2000). A more severe lung injury model or over a longer observation period may have revealed significant differences among the three groups.

All three ventilation strategies demonstrated increases in lung inflammatory mediators and histopathologic markers of injury, although there were no significant differences among the groups. This suggests that the Tween injury resulted in inflammation, but that there were no significant differences in biotrauma or alveolar injury associated with the ventilation method in this acute study. The lack of difference among groups and the relatively high variability at each timepoint may reflect the short duration of the protocol, which did not allow sufficient time for mechanical injury to upregulate inflammatory mediators. Tween was directed to the diaphragmatic lobes, which may be reflected by the increased wet/dry weight, higher histologic injury score, as well as IL-6 expression, which were all relatively higher in the diaphragmatic lung lobes as compared with the apical lung lobes. Although not statistically significant, the V_T_10 group demonstrated relatively higher expression of IL-8, where IL-8 has been affiliated with overdistension (Dreyfuss et al., 2003).

### Limitations

A Tween injury was specifically selected for this study design to maximize applicability as it induces surfactant depletion, which is involved in the pathogenesis of both pulmonary and extrapulmonary ARDS. However, the lungs have a tendency to trend towards recovery after Tween injury, which may limit the ability to distinguish differences among the ventilation treatment strategies by the study end. Furthermore, the duration of the protocol may not have been sufficient to detect meaningful differences in respiratory parameters or inflammatory signals. The study compares two volume-targeted (V_T_6 and V_T_10) modes against a pressure-regulated (APRV) mode. However, APRV does not allow for controlling certain variables, such as tidal volume and driving pressure, that have been associated with VILI. Given this limitation, further study comparing CD-APRV against another pressure-targeted mode as well as manually-directed APRV to verify non-inferiority is warranted. Animals were paralyzed in order to ensure representative measurements were captured for computational analysis. While this is a pilot study, pharmacologic paralysis is not clinically realistic for every mechanically ventilated patient and also eliminates the potential benefit of spontaneous breathing using APRV (Swindin et al., 2020). Though the study was not blinded because of the nature of making mechanical ventilator adjustments, animals were randomized and the data interpretation was performed to mitigate this with the use of blinded histopathology assessment and an independent statistician.

## Conclusions

In this proof-of-concept study using a short-duration animal model, APRV was successfully implemented on a transport ventilator with informed adjustments in T_Low_ based on respiratory mechanics and computational direction. Additionally, the feasibility of developing an empiric approach to ventilation for patients with (or at risk of developing) ARDS using a transport ventilator was demonstrated. This preliminary study demonstrated that computational direction is feasible and poises the development of an automated system with CD integration. CD-APRV demonstrated similar safety when compared to the low tidal volume (V_T_6) strategy but with improved CO_2_ elimination, though it requires further feasibility investigations. Finally, although the CD-APRV was tested on a specific ventilator model, it could be integrated with any ventilator that offers APRV as a mode (Spiegel and Hockstein, 2022).

## Data Availability

The original contributions presented in the study are included in the article/Supplementary Material, further inquiries can be directed to the corresponding author.

## Author disclaimer

The views expressed in this material are those of the authors, and do not reflect the official policy or position of the U.S. Government, the Department of Defense, or the Department of the Army. This study proposed an investigational use of APRV on the ZOLL EMV+ 731 Series transport ventilator, as it is not otherwise configured to deliver APRV. This study was therefore a joint industry/academic venture supported by a U.S. Department of Defense contract (W81XWH-20-1–0696), to both integrate the APRV mode and test CD-APRV.
