# Supplementary material for: Computationally-directed mechanical ventilation in a porcine model of ARDS
Source: Front Physiol. 2025 Nov 26;16:1602578. doi: 10.3389/fphys.2025.1602578 (PMC12689400; doi:10.3389/fphys.2025.1602578)
Supplement: Supplementary file 1 [file Table1.docx]

Supplementary Material – Table 1

**Supplemental Table 1. Quantified Histology Scoring.**

| **Parameter** | **1** | **2** | **3** | **4** |
| --- | --- | --- | --- | --- |
| Microatelectasis | None | Small Regional Areas | Large Regional Areas | Entire Field |
| WBC in Air Space | None | 1-2 Air Spaces | 3-4 Air Spaces | > 4 Air Spaces |
| RBC in Air Space | None | 1-2 Air Spaces | 3-4 Air Spaces | > 4 Air Spaces |
| Edema/Fibrin | None | 1 Air Spaces | 2 Air Spaces | > 2 Air Spaces |
| Capillary Congestion | None | Few Capillaries | 1/2 of the field | Most of the Field |

|  | V_T_6 | | | | | | | | | |
| --- | --- | --- | --- | --- | --- | --- | --- | --- | --- | --- |
|  | Micro-Atelectasis | | WBC in Air Space | | RBC in Air Space | | Edema/Fibrin | | Capillary Congestion | |
|  | Apex | Diaphragm | Apex | Diaphragm | Apex | Diaphragm | Apex | Diaphragm | Apex | Diaphragm |
| Rater 1 | 2.1 ± 0.7 | 2.4 ± 0.9 | 1.2 ± 0.5 | 2.9 ± 1.2 | 1.2 ± 0.6 | 1.6 ± 1.0 | 1.2 ± 0.5 | 1.8 ± 1.2 | 2.0 ± 1.1 | 2.0 ± 1.0 |
| Rater 2 | 2.2 ± 0.9 | 2.5 ± 1.0 | 1.8 ± 1.0 | 3.3 ± 1.0 | 1.2 ± 0.7 | 2.2 ± 1.2 | 1.6 ± 0.9 | 2.5 ± 1.1 | 2.2 ± 1.1 | 2.8 ± 1.0 |
|  |  |  |  |  |  |  |  |  |  |  |
|  | V_T_10 | | | | | | | | | |
|  | Micro-Atelectasis | | WBC in Air Space | | RBC in Air Space | | Edema/Fibrin | | Capillary Congestion | |
|  | Apex | Diaphragm | Apex | Diaphragm | Apex | Diaphragm | Apex | Diaphragm | Apex | Diaphragm |
| Rater 1 | 2.5 ± 0.8 | 2.3 ± 0.9 | 1.2 ± 0.4 | 2.7 ± 1.2 | 1.2 ± 0.6 | 1.5 ± 1.0 | 1.4 ± 0.8 | 1.7 ± 1.0 | 1.3 ± 0.6 | 1.5 ± 0.8 |
| Rater 2 | 2.4 ± 1.0 | 2.0 ± 0.9 | 1.5 ± 0.9 | 3.3 ± 1.1 | 1.2 ± 0.5 | 2.0 ± 1.1 | 1.7 ± 1.0 | 2.3 ± 1.0 | 2.0 ± 1.0 | 3.3 ± 0.7 |
|  |  |  |  |  |  |  |  |  |  |  |
|  | CD-APRV | | | | | | | | | |
|  | Micro-Atelectasis | | WBC in Air Space | | RBC in Air Space | | Edema/Fibrin | | Capillary Congestion | |
|  | Apex | Diaphragm | Apex | Diaphragm | Apex | Diaphragm | Apex | Diaphragm | Apex | Diaphragm |
| Rater 1 | 2.2 ± 0.8 | 2.6 ± 0.7 | 1.3 ± 0.8 | 2.8 ± 1.3 | 1.3 ± 0.8 | 1.7 ± 1.1 | 1.5 ± 1.0 | 2.0 ± 1.3 | 1.9 ± 1.0 | 2.3 ± 0.9 |
| Rater 2 | 1.9 ± 0.9 | 2.2 ± 1.0 | 1.5 ± 1.0 | 2.9 ± 1.1 | 1.2 ± 0.6 | 1.5 ± 1.0 | 1.4 ± 0.8 | 1.8 ± 0.9 | 2.1 ± 0.8 | 3.0 ± 0.8 |
