# Supplementary material for: Computationally-directed mechanical ventilation in a porcine model of ARDS
Source: Front Physiol. 2025 Nov 26;16:1602578. doi: 10.3389/fphys.2025.1602578 (PMC12689400; doi:10.3389/fphys.2025.1602578)

Supplementary Material – Figure 1

**Supplemental Figure 1: CD-APRV Protocol.** Flow chart illustrating the CD-APRV protocol during the animal experiments, including the procedure for T_Low_ titration at each hour.


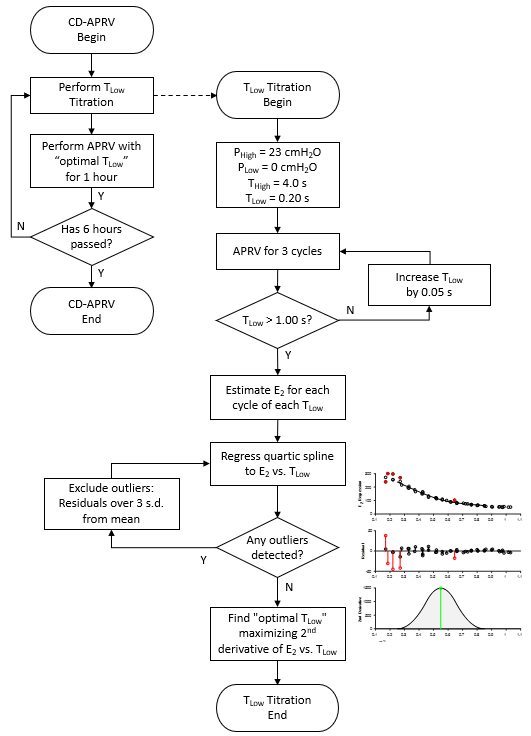

Supplement: Supplementary file 2 [file DataSheet3.docx]
