# Supplementary material for: Computationally-directed mechanical ventilation in a porcine model of ARDS
Source: Front Physiol. 2025 Nov 26;16:1602578. doi: 10.3389/fphys.2025.1602578 (PMC12689400; doi:10.3389/fphys.2025.1602578)
Supplement: Supplementary file 3 [file Table2.docx]

Supplementary Material – Table 2

**Supplemental Table 2. Respiratory Parameters.**

|  |  | BL | T0 | T1 | T2 | T3 | T4 | T5 | T6 |
| --- | --- | --- | --- | --- | --- | --- | --- | --- | --- |
| Peak Pressure (cmH_2_O) | V_T_6 | 19.0 [17.3-21.8] | 16.5 [15.0-23.2] | 21.7 [19.4-26.2] | 22.3 [20.5-26.7] | 23.5 [20.5-26.7] | 23.0 [20.9-26.8] | 23.7 [19.1-26.2] | 23.4 [19.0-26.9] |
|  | V_T_10 | 20.2 [19.3-20.7] | 23.9 [22.9-28.4] | 24.7 [22.4-26.1] | 25.4 [21.4-26.5] | 25.4 [22.5-25.9] | 24.5 [22.4-25.7] | 24.0 [22.6-24.8] | 23.5 [22.5-25.3] |
|  | CD-APRV | 23.0 [22.0-24.6] | 29.8 [29.2-30.2] | 29.5 [29.0-29.9] | 29.4 [29.1-29.7] | 29.4 [28.8-29.7] | 29.4 [28.6-29.7] | 29.5 [29.0-29.6] | 29.2 [28.9-29.4] |
| Plateau Pressure (cmH_2_O) | V_T_6 | --- | --- | --- | --- | --- | --- | --- | --- |
|  | V_T_10 | --- | --- | --- | --- | --- | --- | --- | --- |
|  | CD-APRV | 21.9 [21.0-23.2] | 20.9 [20.4-21.4] | 21.0 [20.1-21.9] | 21.3 [20.8-22.1] | 22.0 [21.5-22.4] | 22.2 [21.8-22.7] | 22.1 [21.8-22.8] | 22.0 [21.8-22.7] |
| Mean Airway Pressure (cmH_2_O) | V_T_6 | 7.4 [7.2-8.3] | 7.7 [6.3-15.2] | 9.1 [7.7-13.0] | 11.2 [8.2-12.2] | 11.2 [8.6-12.5] | 11.4 [8.5-12.8] | 11.3 [8.5-12.8] | 11.3 [8.5-12.7] |
|  | V_T_10 | 7.7 [7.5-8.2] | 7.8 [7.6-8.2] | 8.2 [7.8-9.1] | 8.6 [8.1-9.6] | 8.0 [6.7-8.2] | 8.0 [7.7-8.6] | 8.0 [7.7-8.2] | 7.8 [7.6-8.4] |
|  | CD-APRV | 8.2 [7.9-8.6] | 19.5 [19.3-20.1] | 20.0 [19.6-20.2] | 19.8 [19.6-20.1] | 19.9 [19.8-20.5] | 20.1 [20.1-20.5] | 20.2 [20.1-20.5] | 20.4 [20.1-20.6] |
| PEEP (cmH_2_O) | V_T_6 | 5.0 [5.0-5.0] | 5.0 [5.0-5.0] | 10.0 [5.0-12.0] | 10.0 [8.0-12.0] | 10.0 [8.0-10.0] | 10.0 [8.0-10.0] | 8.0 [6.0-10.0] | 10.0 [8.0-10.0] |
|  | V_T_10 | 5.0 [5.0-5.0] | 5.0 [5.0-5.0] | 5.0 [5.0-5.0] | 5.0 [5.0-5.0] | 5.0 [5.0-5.0] | 5.0 [5.0-5.0] | 5.0 [5.0-5.0] | 5.0 [5.0-5.0] |
|  | CD-APRV | --- | --- | --- | --- | --- | --- | --- | --- |
| Tidal Volume (mL/kg) | V_T_6 | 9.9 [5.9-9.9] | 6.0 [5.9-6.0] | 6.0 [5.9-6.0] | 6.0 [5.9-6.0] | 6.0 [5.9-6.0] | 6.0 [5.9-6.0] | 6.0 [5.8-6.0] | 6.0 [5.8-6.0] |
|  | V_T_10 | 10.0 [9.9-10.1] | 10.0 [9.9-10.1] | 10.0 [9.9-10.1] | 10.0 [9.9-10.1] | 10.0 [9.9-10.1] | 10.0 [9.9-10.1] | 10.0 [9.9-10.1] | 10.0 [9.9-10.1] |
|  | CD-APRV | 9.6 [9.6-10.3] | 9.5 [9.1-10.9] | 10.7 [8.9-10.8] | 9.6 [8.8-10.8] | 10.1 [9.3-10.5] | 10.9 [9.3-11.3] | 10.3 [9.7-11.6] | 10.4 [9.9-11.0] |
| Respiratory Rate (breaths/min) | V_T_6 | 15 [12-18] | 15 [15-18] | 15 [15-24] | 24 [22-30] | 28 [24-30] | 30 [30-32] | 32 [30-34] | 30 [30-32] |
|  | V_T_10 | 14 [12-15] | 12 [12-12] | 12 [12-15] | 15 [12-15] | 12 [12-15] | 12 [12-15] | 13 [12-15] | 12 [12-15] |
|  | CD-APRV | 12 [12-14] | 14 [14-14] | 14 [14-14] | 14 [14-14] | 14 [13-14] | 14 [14-14] | 14 [14-14] | 14 [14-14] |
| Minute Ventilation (L/min) | V_T_6 | 5.0 [3.4-7.0] | 3.0 [2.7-3.6] | 3.3 [2.8-4.0] | 4.7 [3.5-5.6] | 5.2 [4.5-6.1] | 5.9 [5.5-6.6] | 6.2 [5.6-6.7] | 5.9 [5.7-6.5] |
|  | V_T_10 | 5.8 [5.2-6.7] | 4.5 [4.1-5.4] | 4.4 [4.0-6.3] | 4.6 [4.1-5.1] | 4.6 [3.9-5.1] | 4.3 [3.9-5.2] | 4.7 [3.9-5.6] | 5.0 [4.0-5.3] |
|  | CD-APRV | 5.5 [5.1-6.4] | 4.7 [4.1-5.0] | 4.3 [4.1-4.9] | 4.3 [4.1-4.7] | 4.3 [4.1-4.6] | 4.6 [4.4-4.8] | 4.7 [4.5-4.9] | 4.6 [4.2-4.9] |
| pH | V_T_6 | 7.44 [7.42-7.46] | 7.48 [7.40-7.53] | 7.21 [7.15-7.31] | 7.25 [7.22-7.33] | 7.29 [7.23-7.32] | 7.32 [7.29-7.36] | 7.34 [7.34-7.38] | 7.37 [7.34-7.40] |
|  | V_T_10 | 7.43 [7.42-7.46] | 7.49 [7.46-7.52] | 7.40 [7.32-7.45] | 7.41 [7.38-7.44] | 7.39 [7.37-7.44] | 7.43 [7.38-7.46] | 7.44 [7.38-7.47] | 7.45 [7.44-7.46] |
|  | CD-APRV | 7.43 [7.41-7.46] | 7.49 [7.40-7.53] | 7.41 [7.38-7.42] | 7.43 [7.37-7.47] | 7.45 [7.37-7.50] | 7.44 [7.39-7.47] | 7.42 [7.33-7.44] | 7.45 [7.35-7.49] |
| PaO_2_:FiO_2_ Ratio | V_T_6 | 558.3 [489.1-597.1] | 75.3 [62.0-114.9] | 244.0 [149.9-311.8] | 329.0 [104.6-362.5] | 356.8 [272.6-409.5] | 405.5 [328.0-442.8] | 397.3 [379.3-449.6] | 415.5 [383.0-443.4] |
|  | V_T_10 | 567.3 [496.2-604.6] | 85.9 [64.7-120.0] | 336.7 [242.4-368.1] | 316.3 [307.0-370.0] | 335.7 [300.5-378.2] | 339.7 [300.0-406.3] | 358.3 [298.7-406.7] | 353.3 [297.3-397.7] |
|  | CD-APRV | 549.7 [504.8-575.5] | 60.5 [58.4-108.8] | 198.5 [91.6-267] | 311.2 [151.4-395.7] | 359.7 [142.3-380.3] | 311.5 [169.3-396.8] | 291.8 [222.1-402.8] | 316.6 [269.8-362.4] |
| FiO_2_ | V_T_6 | 1.0 [1.0-1.0] | 1.0 [1.0-1.0] | 0.5 [0.4-0.7] | 0.4 [0.4-0.5] | 0.4 [0.4-0.5] | 0.4 [0.4-0.5] | 0.4 [0.4-0.5] | 0.4 [0.4-0.5] |
|  | V_T_10 | 1.0 [1.0-1.0] | 1.0 [1.0-1.0] | 0.8 [0.5-1.0] | 0.4 [0.3-0.8] | 0.4 [0.3-0.5] | 0.3 [0.3-0.4] | 0.4 [0.3-0.4] | 0.4 [0.3-0.4] |
|  | CD-APRV | 1.0 [1.0-1.0] | 1.0 [1.0-1.0] | 1.0 [1.0-1.0] | 1.0 [0.7-1.0] | 0.8 [0.6-1.0] | 0.6 [0.5-0.8] | 0.6 [0.5-0.8] | 0.6 [0.5-0.8] |
| PaCO_2_ (mmHg) | V_T_6 | 39.7 [38.1-41.8] | 30.2 [27.8-43.1] | 74.9 [66.0-90.5] | 64.6 [61.5-70.4] | 66.8 [60.2-82.7] | 62.7 [58.7-66.5] | 59.2 [53.3-60.8] | 59.3 [52.3-60.1] |
|  | V_T_10 | 41.7 [39.3-44.9] | 36.9 [28.1-40.0] | 46.8 [41.4-55.9] | 48.1 [44.7-50.1] | 51.2 [44.9-52.8] | 48.9 [45.6-52.3] | 47.8 [44.8-51.3] | 47.5 [45.3-54.4] |
|  | CD-APRV | 42.9 [40.8-44.1] | 32.8 [30.3-43.9] | 43.4 [39.3-44.6] | 41.9 [32.4-47.6] | 38.8 [29.2-48.1] | 37.8 [32.3-43.0] | 40.8 [34.4-46.8] | 38.5 [32.7-52.2] |
