# Supplementary material for: Computationally-directed mechanical ventilation in a porcine model of ARDS
Source: Front Physiol. 2025 Nov 26;16:1602578. doi: 10.3389/fphys.2025.1602578 (PMC12689400; doi:10.3389/fphys.2025.1602578)
Supplement: Supplementary file 4 [file Table3.docx]

Supplementary Material – Table 3

**Supplemental Table 3. Hemodynamics and Fluid Balance.**

|  |  | BL | T0 | T1 | T2 | T3 | T4 | T5 | T6 |
| --- | --- | --- | --- | --- | --- | --- | --- | --- | --- |
| Heart Rate (beat/min) | V_T_6 | 119 [92-126] | 100 [91-113] | 93 [90-104] | 90 [74-114] | 95 [84-109] | 102 [84-116] | 98 [85-104] | 90 [78-99] |
|  | V_T_10 | 97 [93-104] | 124 [102-124] | 90 [77-95] | 88 [86-102] | 85 [72-95] | 92 [75-94] | 92 [74-113] | 90 [78-96] |
|  | CD-APRV | 104 [99-109] | 123 [121-130] | 115 [99-130] | 109 [94-145] | 94 [90-132] | 99 [87-131] | 103 [80-127] | 105 [78-118] |
| Mean Arterial Pressure (mmHg) | V_T_6 | 125 [111-135] | 119 [110-123] | 117 [104-125] | 122 [112-124] | 122 [111-123] | 114 [108-118] | 105 [104-109] | 99 [93-109] |
|  | V_T_10 | 110 [103-131] | 118 [95-121] | 115 [107-125] | 119 [108-120] | 121 [112-123] | 111 [97-123] | 107 [88-119] | 86 [81-106] |
|  | CD-APRV | 123 [107-138] | 107 [94-118] | 106 [91-120] | 101 [95-110] | 93 [86-105] | 91 [88-94] | 85 [82-97] | 77 [67-87] |
| Pulmonary Artery Pressure (mmHg) | V_T_6 | 26.5 [23.5-29.0] | 41.0 [38.8-46.3] | 33.0 [27.0-39.3] | 36.0 [29.0-36.5] | 35.5 [30.5-38.0] | 32.5 [30.5-36.5] | 31.5 [27.8-36.3] | 30.5 [24.8-36.3] |
|  | V_T_10 | 26.5 [23.0-29.3] | 34.0 [32.3-28.5] | 28.0 [25.3-30.0] | 30.5 [29.3-31.8] | 27.0 [23.8-28.5] | 26.5 [24.5-28.3] | 26.0 [23.0-28.0] | 25.5 [24.0-26.3] |
|  | CD-APRV | 32.0 [24.5-39.0] | 30.0 [28.0-39.5] | 30.0 [27.0-36.5] | 32.0 [27.5-38.5] | 28.0 [27.0-37.5] | 34.0 [30.5-35.5] | 31.0 [30.5-34.5] | 29.0 [26.5-33.5] |
| Pulmonary Capillary Wedge Pressure (mmHg) | V_T_6 | 13.0 [12.3-13.8] | 15.0 [11.0-17.5] | 16.0 [13.5-17.5] | 16.0 [13.0-19.0] | 15.0 [12.5-18.5] | 15.0 [12.5-16.5] | 14.0 [12.5-16.5] | 14.0 [12.5-16.5] |
|  | V_T_10 | 12.5 [9.8-16.0] | 12.5 [10.5-14.8] | 13.0 [11.0-16.0] | 13.5 [12.0-15.0] | 12.5 [10.8-13.5] | 11.5 [11.0-13.5] | 12.5 [11.0-14.0] | 12.0 [11.0-12.3] |
|  | CD-APRV | 13.0 [11.8-14.5] | 15.5 [13.5-19.0] | 15.5 [13.5-16.8] | 18.0 [14.8-19.8] | 17.0 [13.8-18.8] | 16.5 [14.5-17.0] | 15.0 [15.0-19.5] | 15.0 [15.0-15.8] |
| Cumulative Fluids (L) | V_T_6 | 1.28 [1.28-1.28] | 1.57 [1.53-1.58] | 1.81 [1.72-1.89] | 1.99 [1.88-2.05] | 2.13 [2.03-2.17] | 2.20 [2.15-2.30] | 2.35 [2.15-2.42] | 2.49 [2.22-2.54] |
|  | V_T_10 | 1.28 [1.28-1.28] | 1.55 [1.54-1.58] | 1.71 [1.65-1.74] | 1.84 [1.75-1.88] | 1.96 [1.85-2.03] | 2.09 [1.95-2.19] | 2.22 [2.05-2.35] | 2.35 [2.15-2.51] |
|  | CD-APRV | 1.28 [1.28-1.28] | 1.58 [1.55-1.58] | 1.70 [1.70-1.83] | 1.82 [1.79-1.95] | 2.05 [1.91-2.10] | 2.16 [2.01-2.20] | 2.28 [2.13-2.30] | 2.39 [2.24-2.41] |
| Cumulative Urine Output (mL) | V_T_6 | 130 [145-225] | 167 [73-270] | 227 [210-485] | 512 [460-692] | 597 [525-715] | 773 [675-830] | 875 [720-982] | 955 [720-1207] |
|  | V_T_10 | 100 [50-150] | 140 [130-225] | 310 [240-415] | 465 [448-600] | 715 [520-768] | 890 [710-1068] | 975 [830-1215] | 1165 [945-1315] |
|  | CD-APRV | 140 [100-210] | 240 [188-275] | 310 [260-385] | 435 [325-519] | 515 [365-537] | 565 [408-637] | 640 [433-680] | 694 [495-705] |
| Fluid Balance (L) | V_T_6 | 1.17 [1.03-1.23] | 1.43 [1.29-1.51] | 1.44 [1.37-1.67] | 1.50 [1.28-1.59] | 1.51 [1.39-1.58] | 1.47 [1.21-1.63] | 1.47 [1.12-1.69] | 1.47 [1.13-1.74] |
|  | V_T_10 | 1.18 [1.13-1.21] | 1.38 [1.36-1.45] | 1.33 [1.28-1.47] | 1.26 [1.24-1.38] | 1.30 [1.21-1.31] | 1.20 [1.18-1.29] | 1.22 [1.15-1.38] | 1.21 [1.12-1.34] |
|  | CD-APRV | 1.14 [1.07-1.18] | 1.32 [1.22-1.37] | 1.36 [1.30-1.56] | 1.39 [1.35-1.65] | 1.57 [1.41-1.74] | 1.67 [1.43-1.81] | 1.73 [1.47-1.91] | 1.75 [1.54-2.01] |
