# Supplementary material for: Computationally-directed mechanical ventilation in a porcine model of ARDS
Source: Front Physiol. 2025 Nov 26;16:1602578. doi: 10.3389/fphys.2025.1602578 (PMC12689400; doi:10.3389/fphys.2025.1602578)
Supplement: Supplementary file 5 [file DataSheet2.docx]

Supplementary Material – Methods 2

**Supplemental Methods 2: Power Analysis, Model Justification, and Animal Care**

**Power Analysis**

The selection of sample size was based on the analysis of PaO_2_:FiO_2_ ratio as the primary endpoint. For simplicity, sample size was calculated using a two-sample Satterthwaite t-test for mean differences. Bonferroni-adjusted significance level was used in the calculation to account for the intended Tukey-Kramer adjustment in the analysis. Estimates of the mean and standard deviation of the PaO_2_:FiO_2_ ratio were calculated from previous studies and used in the sample size calculation (1, 2). For these calculations the estimated mean ± standard deviation of the 6mL/kg group were 205.18 ± 48.43 mmHg, and 114.61 ± 43.39 mmHg in the 10mL/kg group. The calculation for the two-sample approximation resulted in a sample size of 9 pigs per group, allowing us to detect a difference of this size with at least 90% power. Animals were randomized to ventilator strategy using the ranuni function in SAS (V9.4).

**Animal and Model Justification**

**Pig Model:** The pig is an excellent model for lung injury because pigs have similar pulmonary anatomy, physiology, and inflammatory response as humans. The size of pig lungs mimic that of a human and allows for the application of human ventilators and settings to maximize clinical applicability. The proposed 6-hour study of surfactant wash-out has been previously validated (1). The use of a bronchoscope allows for precise direction of the Tween into the dependent basilar lung regions (3). The Tween surfactant washout model is suited to test the proposed hypothesis as it generates the surfactant disruption and heterogeneous injury that is associated with many forms of ARDS (4, 5). Thus, the model recapitulates the progression of injury that occurs in humans including clinical features and physiological, biological, and pathological changes, as recommended for a translational model (6).

**Small Animal Model:** The use of a small animal model would limit the clinical application of the results of the studies as there is a marked difference in lung structure between rodents and humans (7). Smaller animals tend to have a more compliant chest wall and respond disparately to lung injury models (7) in which loss of chest wall compliance contributes to the progression of injury (6). Finally, the use of a smaller animal would not allow treatment with similar monitoring and protocols that a human patient would receive in an intensive care setting, further limiting translation of the results of the model.

**Alternative Models:** The proposed animal experiments cannot be duplicated in an in vitro system as these would not adequately mimic the complex and multifactorial progression of lung injury to provide results that would inform human use.

**Housing, Husbandry, and Monitoring**

Pigs were transported from a USDA-approved farm to the Department of Laboratory Animal Resources (DLAR) at SUNY Upstate Medical University. They were provided chow and water ad lib until the night before the procedure, at which time they were made NPO. Pigs were housed in pens that were cleaned twice daily. They were provided enrichment activities including marshmallows, popsicles, and balls. The DLAR staff would regularly play with the pigs, and pigs were allowed to cohabitate with other pigs. They were allowed a 7-day acclimatization period prior to experiments.

All animal experiments were conducted at SUNY Upstate Medical University. Animals were pretreated with atropine (0.04 mg/mL), tiletamine hydrochloride and zolazepam (5mg/kg) and xylazine (2mg/kg) intramuscularly. An IV catheter was placed into an ear vein for continuous infusion of ketamine (9mg/mL) / xylazine (0.009mg/mL) and continued through a central venous line, once placed. Animals were continuously monitored for anesthesia adequacy ensuring a Stage III, Plane 2 level of anesthesia. All animals were humanely euthanized at the study end point (6 hours after injury) or if they were found to no longer have a survivable injury, as determined by refractory septic shock (unresponsive to fluids and maximal vasopressor support), or refractory acute respiratory distress syndrome (persistent PaO_2_:FiO_2_ < 50 mmHg despite maximal ventilatory support) with a pentobarbital (150 mg/kg) overdose. Only one animal in the V_T_6 group had an early death four hours after injury from sudden cardiovascular collapse that was not responsive to epinephrine injection. There were no other adverse events, and no animals were excluded from the study or analysis. Animals were monitored continuously for hemodynamic and respiratory status, with hourly recordings of associated parameters and arterial blood gas measurements.

**References**

1. Ramcharran H, Bates JHT, Satalin J, Blair S, Andrews PL, Gaver DP, et al. Protective ventilation in a pig model of acute lung injury: timing is as important as pressure. J Appl Physiol (1985). 2022;133(5):1093–105.

2. Jain SV, Kollisch-Singule M, Satalin J, Searles Q, Dombert L, Abdel-Razek O, et al. The role of high airway pressure and dynamic strain on ventilator-induced lung injury in a heterogeneous acute lung injury model. Intensive Care Med Exp. 2017;5(1):25.

3. Judge EP, Hughes JM, Egan JJ, Maguire M, Molloy EL, O'Dea S. Anatomy and bronchoscopy of the porcine lung. A model for translational respiratory medicine. Am J Respir Cell Mol Biol. 2014;51(3):334–43.

4. Matute-Bello G, Downey G, Moore BB, Groshong SD, Matthay MA, Slutsky AS, et al. An official American Thoracic Society workshop report: features and measurements of experimental acute lung injury in animals. Am J Respir Cell Mol Biol. 2011;44(5):725–38.

5. Slutsky AS, Ranieri VM. Ventilator-induced lung injury. N Engl J Med. 2013;369(22):2126–36.

6. Matute-Bello G, Frevert CW, Martin TR. Animal models of acute lung injury. Am J Physiol Lung Cell Mol Physiol. 2008;295(3):L379–99.

7. Irvin CG, Bates JH. Measuring the lung function in the mouse: the challenge of size. Respiratory research. 2003;4:4.
