# Supplementary material for: Computationally-directed mechanical ventilation in a porcine model of ARDS
Source: Front Physiol. 2025 Nov 26;16:1602578. doi: 10.3389/fphys.2025.1602578 (PMC12689400; doi:10.3389/fphys.2025.1602578)
Supplement: Supplementary file 6 [file DataSheet1.docx]

Supplementary Material – Methods 1

**Supplemental Methods 1 - Titration of Expiratory Duration (T_Low_) During Airway Pressure Release Ventilation**

The ZOLL EMV+ 731 Series ventilator was controlled by a MATLAB program (MathWorks, Natick, MA), for automated titration of T_Low_ to determine the optimal computationally-directed TCAV (CD-APRV) settings. To obtain real-time adjustments of CD-APRV using the EMV+ 731 Series, we relied on an inverse modeling approach to characterize global respiratory system mechanics during positive pressure ventilation based on the nonlinear equation of motion (1, 2, 3):

$P=R_{1}\dot{V+}R_{2}\left| \dot{V} \right|\dot{V}+E_{1}V+E_{2}V^{2}+P_{0}$ (S-1)

where *P* denotes airway pressure, *V* is volume and $\dot{V}$ is flow. The model parameters account for linear and nonlinear resistive (*R*_1_ and *R*_2_) and elastic (*E*_1_ and *E*_2_) properties, as well as non-zero end-expiratory pressure (*P*_0_). The *E*_1_ and *E*_2_ parameters denote the volume-independent and volume-dependent components of elastance, which allows for the determination of the percentage of nonlinearity in elastic pressure:


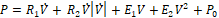


$\%E_{2}=100\%\times\frac{E_{2}V}{E_{1}+E_{2}V}$ (S-2)

Since %*E*_2_ quantifies the deviation of linearity between elastic pressure and volume, it can provide quantitative information on the degree of intratidal recruitment vs. overdistention occurring during ventilation. Based on previous modeling and imaging studies, positive values of %*E*_2_ may reflect overdistention in the parenchyma, while negative values of %*E*_2_ may reflect intratidal recruitment / derecruitment (4, 5, 6, 7).

To computationally-direct the APRV settings during ventilation, we estimated all parameters from the nonlinear equation of motion using multiple linear regression (8) over a range of T_Low_ (i.e., a “T_Low_ titration”). We then fit a 4^th^ order spline to the *E*_2_ vs. T_Low_ curve (Figure S1A), from which we determine the so-called “optimal” T_Low,_ based on the second derivative of the curve (Figure S1C). The local maximum of the second derivative for this curve corresponds to a “knee” in the *E*_2_ vs. T_Low_ curve. Given that *E*_2_ reflects the nonlinear processes of derecruitment or overdistention, its second derivative will indicate the value of T_Low_ above which minimal changes in these processes will occur, despite increasing T_Low_.

Figure S1. (A) Example *E*_2_ vs. T_Low_ data, obtained during T_Low_ titration, with corresponding spline fit; (B) corresponding residuals between actual *E*_2_ values and spline prediction. Red symbols denote outliers omitted from the fit due to high variation of residuals; (C) second derivative of spline fit, yielding a maximal value at the “optimal” T_Low_.

**References**

1. Kaczka DW HJ, Hawley ML. Physiologic and Medical Assessments of Respiratory Mechanics and Ventilation. Biomedical Engineering of Pancreatic, Pulmonary, and Renal Systems, and Applications to Medicine: Elsevier Inc; 2023. p. 359-414.

2. JHT B. The linear single-compartment model. Lung Mechanics: And Inverse Modeling Approach: Cambridge University Press; 2009.

3. Bates JHT, Kaczka DW, Kollisch-Singule M, Nieman GF, Gaver DP. Atelectrauma can be avoided if expiration is sufficiently brief: evidence from inverse modeling and oscillometry during airway pressure release ventilation. Critical care. 2024;28(1):329.

4. Kaczka DW, Chitilian HV, Vidal Melo MF. Respiratory Monitoring. In: Gropper M, Eriksson L, Fleisher L, Wiener-Fronish J, Cohen N, Leslie K, editors. Miller's Anesthesia. 9th ed. Philadelphia. PA: Elsevier; 2020. p. 1298-339.

5. Kano S, Lanteri CJ, Duncan AW, Sly PD. Influence of nonlinearities on estimates of respiratory mechanics using multilinear regression analysis. J Appl Physiol (1985). 1994;77(3):1185-97.

6. Carvalho AR, Spieth PM, Pelosi P, Vidal Melo MF, Koch T, Jandre FC, et al. Ability of dynamic airway pressure curve profile and elastance for positive end-expiratory pressure titration. Intensive Care Med. 2008;34(12):2291-9.

7. Amini R, Herrmann J, Kaczka DW. Intratidal overdistention and derecruitment in the injured lung: A simulation study. IEEE Trans Biomed Eng. 2017;64(3):681-9.

8. Kaczka DW, Barnas GM, Suki B, Lutchen KR. Assessment of time-domain analyses for estimation of low-frequency respiratory mechanical properties and impedance spectra. Ann Biomed Eng. 1995;23:135-51.
